# Evaluation of Sleep Patterns and Self-Reported Academic Performance among Medical Students at the University of Ghana School of Medicine and Dentistry

**DOI:** 10.1155/2019/1278579

**Published:** 2019-06-11

**Authors:** Henry Jeremy Lawson, Jude Tettey Wellens-Mensah, Salamatu Attah Nantogma

**Affiliations:** ^1^Department of Community Health, College of Health Sciences, P.O. Box KB 4236, University of Ghana, Ghana; ^2^Department of Medicine and Therapeutics, P.O. Box KB 4236, Korle Bu Teaching Hospital, Accra, Ghana; ^3^Maamobi General Hospital, Private Mail Bag, Accra, Ghana

## Abstract

**Background:**

Sleep habits and problems play a vital role in determining sleep quality. We describe sleep habits and problems among medical students and assess their possible effect on self-reported academic performance.

**Methods:**

We conducted a cross-sectional study among medical students at the University of Ghana during the 2014/2015 academic year. Data was collected using the Pittsburgh Sleep Quality Index (PSQI), a self-report questionnaire that assesses sleep quality over a 1-month time interval.

**Results:**

153 medical students were recruited comprising 83 (54.2%) females and 70 (45.8%) males with a mean age of 23.1 ± 2.4 years. The mean duration of night sleep was 5.7 ± 1.2 hours; 88 (57.5%) students had sleep latency of 10-30 minutes while 18 (11.8%) woke up nightly. 23 (15%) students experienced nightmares, 13 (8.5%) snored at night, and only one student reported coffee intake of 2-3 times daily. Sleep quality was poor in 86 (56.2%) and was significantly associated with sleep latency, morning tiredness, daytime sleepiness during lectures, academic performance, living conditions, leisure time, frequency of nocturnal awakenings, waking up due to noise, sleep walking, and nocturnal awakening to use washroom. There was also a significant positive relation between sleep quality and academic performance (X^2^ = 10.004 p = 0.019).

**Conclusion:**

Poor sleep quality and daytime dysfunction are widespread among medical students in Ghana. There was a significant positive relation between sleep quality and self-reported academic performance.

## 1. Introduction

Sleep is a physiological process essential to humans and their normal functioning. Sleep habits and problems are also influenced by physical, mental, and environmental factors such as age, gender, job, lifestyle, emotional tension, and noise (Irish et al, 2007). Adults require on average between* seven and nine hours of sleep each night*. Both the quantity and quality of sleep play an important role in an individual's psychological and physical well-being [1]. During sleep, the brain conducts memory consolidation and integration; adequate and quality sleep eliminates concentration difficulties [2] without which, judgments, mood, and ability to learn and retain information are weakened. [3]. Sleep also allows the brain to better process new experiences and knowledge which increases understanding and retention [4].

### 1.1. Sleep in University Students

Studies conducted on sleep indicate that one-third of adults suffer from sleep problems and that students* sleep less compared to the general population* because of academic stress [5]. University students are particularly susceptible to these increasing demands on sleep [6]. Poor sleep quality has been found to be associated with high failure rates and poor academic performance [7, 8]. Schleider and Günter showed that 54.1% of German university students reported sleep deprivation and poor sleep quality as a cause for their learning and working problems. Students with poor academic performance spend more hours at night reading and often deprive themselves of sleep in a bid to improve their grades. This creates a vicious cycle that is associated with an adverse effect on sleep quality and mental health. Sleep quality and duration is affected by factors such as age, gender, and lifestyle [9, 10]. Female students have been shown to have a higher risk of poor sleep quality [11] and longer* mean total sleep time* [9]. Lifestyle factors such as diet (heavy meals, tyramine/tryptophan rich foods), alcohol, caffeine, drug use, and exercise may contribute to sleep disturbances.

### 1.2. Sleep in Medical Students

Medical students go through long and intensive academic years before becoming physicians. In addition, they face challenging facets of their lives such as family and relationships which affect them psychologically. Psychological stress is a known triggering factor for insomnia and has a bidirectional association with poor sleep quality [12].

There is paucity of data on sleep habits and its problems among student populations in West Africa [3]. The general aim of this study was to examine sleep habits and problems in medical students of UGSMD and assess their possible effect on self-reported academic performance.

*Sleep deprivation and poor sleep quality are particularly prevalent among medical students as seen in studies by Eller et al*., 2006 [13]; Ghanei et al., 2011 [14]; Ghoreishi et al., 2008 [5]; and Eslami et al., 2012 [15]. These studies report that between 43% and 88% of students of the medical sciences suffer from poor sleep quality due to the constant stress and anxiety from their extensive medical curricula, frequent examinations, and fear of failure [16]. Poor sleep quality among medical students usually results in sleep deprivation which manifests as excessive daytime sleepiness [17]. In such students, sleep may be voluntarily sacrificed due to various academic and social commitments or involuntarily curtailed because of a sleep problem. Sleep problems have been found to be associated with increased prevalence of social problems as well as various somatic and/ or psychiatric disorders [18]. Chronic sleep deprivation resulting in poor sleep quality may affect the cognitive and psychomotor performance of medical students. In Africa, few studies in Sudan, Ethiopia, Nigeria, and Ghana ([3, 19–21], respectively) have produced conflicting conclusions on the subject. These studies have used sample sizes ranging from 31 to 6011 medical students. In the Ghanaian study, although medical students had sleep deprivation, they did not experience any physical symptoms.

### 1.3. Study Design and Setting

This descriptive cross-sectional study was conducted at the University of Ghana School of Medicine and Dentistry (UGSMD) during the 2014/2015 academic year. Established in 1962, UGSMD is the first medical school in the country and currently has a student population of 1,118. It runs two medical degree programs: a four-year graduate entry medical programme (GEMP) and the traditional six-year programme. The GEMP consists of one and a half years of preclinical studies and two and a half years of clinical studies. The six-year medical programme consists of three years each of preclinical studies and clinical studies. Second- and third-year preclinical students are shuttled from the University's Legon campus to Korle Bu (a distance of about 19km which is covered in approximately 75 minutes) and back each day, to attend classes. This requires them to wake up early in the morning, further adding to their academic stress. Clinical students serve as subinterns in the various departments in the Korle Bu Teaching Hospital (KBTH) and have to go through various clinical rotations including work on the wards. This sometimes requires them to remain in the hospital until late at night, depriving* themselves* of sleep.

Medical and dental students from both the preclinical and clinical years who gave written consent were conveniently sampled. Students using sedative medications or narcotics for any acute or chronic medical condition were excluded from the study.

## 2. Methods

Data were collected using a 39-item questionnaire adopted from Sweileh* et al.* 2011, and based on the Diagnostic and Statistical Manual of Mental Disorders IV (DSM-IV) criteria for sleep disorders and the Pittsburgh Sleep Quality Index (PSQI) [22]. Students were asked to limit their responses to incidents that occurred during the past one month. The questionnaire consists of six sections covering the following domains: demographic characteristics (gender, age, programme, entry type, academic level, and hostel of residence); sleep habits (time of going to bed, hours of sleep, time of waking, nocturnal intake of coffee, and use of sleeping pills); Sleep problems (time taken to fall asleep, number of times one wakes up the night, snoring, and reasons for failure to maintain sleep); parasomnia (sleep-talking, sleep-walking, and nightmares); daytime tiredness and sleepiness (feeling tired in the morning, daytime sleepiness including sleepiness during lectures and free time, and daytime naps); and general subjective questions (feeling about sleep quality; sleep quality on the night before an exam; academic achievement; feeling about leisure time and living conditions).

The PSQI questionnaire has 19 self-rated questions, which generate seven composite scores. The results give numbers in seven categories: Subjective sleep quality, sleep latency, sleep duration, habitual sleep efficiency, sleep disturbances, use of sleep medication, and daytime dysfunction. The PSQI has good psychometric properties and has been validated among many student populations worldwide including West Africa [23]. As in other populations, a global sum of >5 was indicative of poor sleep quality [22].

### 2.1. Statistical Analyses

For the purposes of our study, sleep habits were defined as time to bed, time to rise, drinking coffee at night, duration of night sleep, and consumption of sleeping pills. Sleeping problems were defined as those which negatively affect an individual's overall schedule of sleep and waking times, to the extent that they interfere with the person's normal physical, mental, and emotional functioning. Chi-square was used to test for significant association between categorical variables and Spearman rank order was used to test for correlation between continuous and ordinal variables. A p-value of < 0.05 was accepted as statistically significant.

## 3. Results

161 students were invited to participate in the study of which 153 (95%) gave written consent and completed the questionnaire. There were more female respondents (83/153, 54.2%) than male respondents (70/153, 45.8%). The students ranged in age from 18 to 30 years (mean 23.1 ± 2.4 years). There were 122 (79.9%) students from the traditional programme and 31 (20.3%) from the GEMP. Preclinical year students represented 57 (37.3%) of the sample while clinical year students represented 96 (62.7%). Of the respondents, 82 (53.6%) resided on the Korle-Bu campus, 49 (32%) were non-resident, whilst 22 (14.4%) resided on the main university campus in Legon (Table 1).

### 3.1. Sleep Characteristics

**Figure 1:**
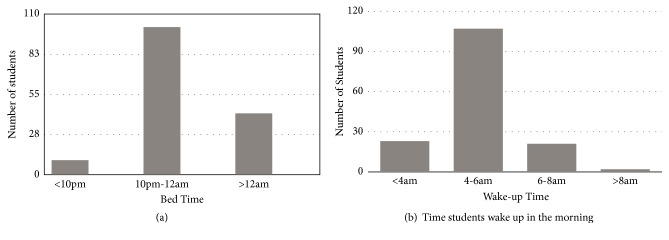


**Figure 2:**
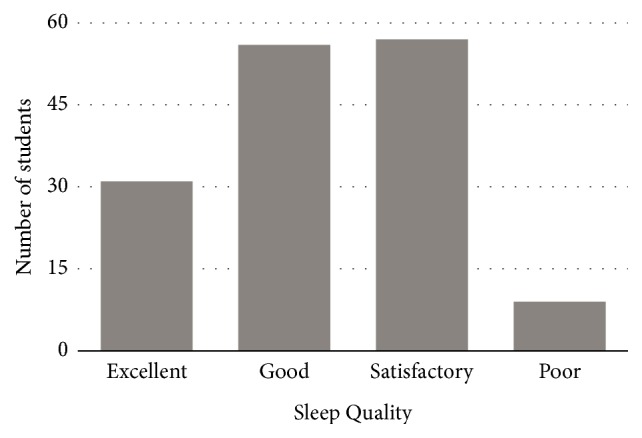
Self-reported quality of sleep.

**Figure 3:**
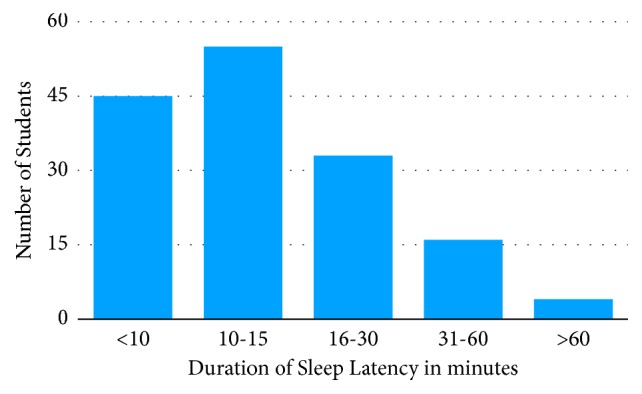
Sleep latency of medical students.

Of the respondents, 10 (6.5%) students went to bed before 10pm, 101 (66%) went to bed between 10pm and midnight; and 42 (27.5%) went to bed after midnight (Figure 1(a)). The average duration of sleep reported by students was 5.7 ± 1.2 hours. Most of the respondents (107, 69.9%) woke up between 4am and 6am and only 2 (1.3%) students woke up after 8am (see Figure 1(b)). Seven students, representing 4.6%, reported using medications to enhance their sleep. A total of 125 (81.7%) students reported that they never drank coffee at night. Sleep latency of less than 10 minutes was reported by 45 (29.4%) of the students: 10-15 minutes was reported by 55 (35.9%) students; 31-60 minutes was reported by 16 (10.5%) students; and only 4 (2.6%) had a sleep latency of more than 60 minutes (Figure 3). Waking up to use the washroom was reported as the major cause of sleep interruption at night. Of the respondents, 110 (71.9%) students woke up at night to use the washroom. Regarding self-reported sleep quality, only 9 (5.9%) of respondents reported poor sleep quality. Thirty-one (20.3%) reported excellent and 56 (36.6%) reported good while 57 (37.3%) reported satisfactory (Figure 2). However, an evaluation of sleep quality using PSQI and 86 (56.2%) had scores of >5, indicative of poor sleep quality.

Spearman Order Rank test was carried out between Global PSQI score and sleep problems (Table 3)* and it* demonstrated that there was significant positive correlation between sleep quality and the following: sleep latency (r = 0.44; p ≤ 0.001), morning tiredness (r = 0.44; p ≤ 0.001), daytime sleepiness during lectures (r = 0.20; p = 0.01), self-reported academic performance (r = 0.26; p ≤ 0.001), living conditions (r =0.33; p ≤ 0.001), leisure time (r = 0.38; p ≤ 0.001), frequency of nocturnal awakenings (r = 0.23 p ≤ 0.001), waking up due to noise (r = 0.28 p ≤ 0.001), sleep walking (r = 0.24; p ≤ 0.001), and nocturnal awakening to use washroom (r = 0.20; p ≤ 0.01). No statistically significant correlation was found between sleep quality and rest of the sleep problems assessed.

## 4. Discussion

### 4.1. Sleep Habits

This study sought to investigate the time students went to bed at night and* woke* up in the morning (duration of* their* night sleep). Majority of students representing 66% of the sample went to bed between 10pm and 12am and 85% of students woke up before 6am. This was consistent with similar studies among medical students in Europe and the Middle East where about 70% of students went to bed between 10pm – 12am [24]. In Africa, a study on sleep practices among medical students in the paediatrics department of University of Nigeria Teaching Hospital, Enugu, showed that the median time students went to bed was 11 pm and more than half of the students woke up before 6am [21]. In another study by Reid and Baker in South Africa, results showed that black students went to bed significantly later than white and Asian students and woke up earlier than white students. Consequently, they were more likely to have a shorter time in bed than white students [25].

These findings generally show that medical students worldwide sleep before but close to midnight and wake up earlier in comparison to the general population. This is* probably* due to the busy nature of their academic schedule which involves learning clinical skills and adjusting to an ever-changing hospital environment resulting in students getting tired by the close of the day.

The current study also showed that the average duration of night sleep among students was 5.7 ± 1.2 hours which is similar to a study conducted among medical students at the University of Cape Coast in Ghana in which mean sleep duration was 5.9 ±1.0 hours [3]. In other studies in Kenyan and Nigerian Universities majority of medical students slept for between 5 and 6 hours at night [21, 23, 26]. Medical students of the University of Ghana had less than 7 hours of sleep which is inadequate as compared to adequate sleep duration which is cited as 7-9 hours in literature [27]. A vast majority of students never drank coffee or took sleep enhancing medication. This is comparable with studies in the Middle East [17, 28] and Africa [29] where coffee intake and sleep enhancing medications by the general population is generally low. [24].

### 4.2. Sleep Problems 

The results pertaining to sleep latency showed that it took 57.5% of students 10-30 minutes to sleep after getting into bed and another 13.1% of students more than 30 minutes to* fall asleep*. This is comparable to a similar study conducted among German undergraduate students [30]. The normal sleep latency among university students and the general population has been reported to be 8.2 minutes [31] and 7 minutes [32], respectively. From these findings it appears it takes the majority of medical students a longer duration to fall asleep as compared to both the nonmedical university student population and the general populace. Once again, the abnormally long sleep latency may be due to academic stress, fear of failure, and inability to cover academic work scheduled for the day. This is more pronounced when examinations are in the offing. There was also a statistically significant positive correlation between sleep quality and sleep latency [28].

*It was observed that 62*%* of students woke up at least once during the night mostly to use the washroom*. From the present study only 8.5% of the population of medical students reported snoring at night. This however was not significantly associated with sleep quality as noted by Sweileh et al., 2011. Snoring occurs in the presence of obstructive sleep apnoea and may lead to poor sleep quality [33]. There was a significant positive correlation between sleep quality and the following: frequency of nocturnal awakenings, waking up due to nocturnal noise, and waking up at night to use the washroom. This was consistent with a study among Palestinian students [28] which also demonstrated that poor sleep quality was associated with more frequent nocturnal awakenings, nocturnal noise, and waking up at night to use the washroom.

### 4.3. Parasomnias

Nightmares were the commonest parasomnias with about 15% of students reporting that they had nightmares at least once in the past month (Table 2). This is consistent with the findings of the American Academy of Sleep Medicine that nightmares are more common as compared to sleep-walking and sleep-talking. From Table 2, only 9.3% and 5.2% of students reported of sleep-talking and sleep-walking, respectively, at least once in the past month which was comparable to findings in other studies [28, 34] and correlation results, as seen in Table 3, showed that poor sleep quality was associated with high frequency of sleep-walking. These abnormal sleep behaviours consist of arousals from deep nonrapid eye movement sleep and are characterized by intense behavioural manifestations of fear and autonomic hyperactivity [34] which in this case may be due to sleep deprivation from abnormal sleep habits and problems.

### 4.4. Excessive Daytime Sleepiness and Morning Tiredness

Results from the present study (Table 2) revealed that excessive sleepiness during the day and morning tiredness was a common experience among medical students. Excessive daytime sleepiness and morning tiredness is usually due to sleep deprivation [35, 36]. This study supports this finding, in that approximately 78% of students experienced daytime sleepiness both during lecture and free times and over 80% of medical students had daytime naps at least once in the past month and experienced morning tiredness. This supports earlier results showing students of the University of Ghana School of Medicine and Dentistry were generally sleep deprived based on their mean duration of sleep of 5.7 hours which is inadequate. The high prevalence of daytime sleepiness among students in this study is consistent with so many other studies among university students in general [37] and medical students worldwide [17, 38–40]. Increasing frequency of morning tiredness and daytime sleepiness during lectures was associated with poor sleep quality according to correlation results from this study (Table 3). Morning tiredness and daytime sleepiness during lectures may affect concentration levels during lecture times and optimum output levels contributing to poor academic performance.

### 4.5. Sleep Quality

*The comparison between* self-reported sleep quality and sleep quality as measured by the Pittsburgh Sleep Quality Index (Table 4) showed that only 5.9% of students* admitted to* poor sleep quality while approximately 56% of students actually had poor sleep quality. The Pittsburgh Sleep Quality Index has been shown to be a reliable tool for assessing sleep quality among adolescent-aged subjects and students [23, 41]. The Pittsburgh Sleep Quality Index demonstrated that poor sleep quality was common among the student population and was well within rates (11.5 - 60%) reported in other environments [42, 43].

### 4.6. Academic Performance

Approximately 30% of students evaluated their academic performance as satisfactory or poor (Table 4). The study null hypothesis was that sleep quality had no association with academic performance of students of the University of Ghana School of Medicine and Dentistry. A Chi square test which was run to test the hypothesis (Table 4) showed there was a significant relationship between quality of sleep and academic performance since p-value was 0.019. Approximately 63.3% of students with good quality of sleep had excellent academic performance and none of the students with good quality of sleep reported poor academic performance. Various studies have shown that many undergraduate students are at risk* of* abnormal sleep habits which predispose them to sleep problems. This poses a further exposure to poor academic performance [44].

### 4.7. Limitations

The greatest limitation to this study was the use of self-reported academic performance instead of actual academic performance. This study was conducted in one of the five medical schools in Ghana and results cannot be extrapolated to cover the country.

## 5. Conclusion

Good quality sleep is essential for general well-being and optimum functioning of all organ systems. In the present study, sleep habits among the students were largely comparable to that of medical students worldwide. Irregular sleep habits due to academic commitments and busy schedules had a grave impact on sleep-wake cycles of students resulting in sleep deprivation and poor sleep quality. Sleep deprivation in the majority of students was characterised by short duration of night sleep, morning tiredness, excessive daytime sleepiness, and frequent daytime naps. There was also a significant relationship between sleep quality and academic performance. Education of medical students in Ghana on sleep problems and practice of good sleep hygiene is recommended. Secondly, evaluation of sleep problems in medical students with poor academic performance should be routinely practiced. Future studies should investigate the effect of sleep habits and problems of medical students on their general health and explore other factors which might affect their sleep habits such as sleep hygiene, watching television, and use of the internet just before going to bed.

## Figures and Tables

**Table 1:** Demographic characteristics of the study population.

Characteristic	Frequency (Total=153)	Percentage (%)
*Gender*		
Male	70	45.8
Female	83	54.2

*Programme*		
Medicine	114	74.5
Dentistry	39	25.5

*Entry*		
Regular	122	79.7
Graduate Entry	31	20.3

*Year of study*		
Pre-clinical year	57	37.3
Clinical year	96	62.7

*Residential status*		
Korle-Bu campus	82	53.6
Non-Resident	49	32.0
Legon campus	22	14.4

**Table 2:** Sleep patterns and frequency of occurrence.

Variable	Frequency (Percentage)				
	Never	< Weekly	Nightly	1-2 nights/week	3-4 nights/week
*Cause of Nocturnal waking*					

Nightmares	130 (85%)	18 (11.8%)	0 (0%)	3(2.0%)	2(1.3%)
Noise	71 (46.4%)	43 (28.1%)	4 (2.6%)	27 (17.6%)	8 (5.2%)
To use washroom	43 (28.1%)	39 (25.5%)	11 (7.2%)	42 (27.5%)	18 (11.8%)

*Frequency of Nocturnal Wakening*					

	58 (37.9%)	38 (24.8%)	18 (11.8%)	21 (13.7%)	18 (11.8%)

*Types of Parasomnias*					

Nightmares	130 (85%)	18 (11.8%)	0 (0%)	3 (2%)	2 (1.3%)
Sleep-talking	138 (90.2%)	12 (7.8%)	0 (0%)	1 (0.7%)	2 (1.3%)
Sleep-walking	145 (94.8%)	8(5.2%)	0 (0%)	0 (0%)	0 (0%)

	Never	<Daily	Daily	1-2days/week	3-4days/week

Morning tiredness	25 (16.3%)	56 (36.6%)	9 (5.9%)	34 (22.2%)	29 (19.0%)
DST during lectures	33 (21.6%)	45 (29.4%)	15 (9.8%)	42 (27.5%)	18 (11.8%)
DST during free time	33 (21.6%)	47 (30.7%)	16 (10.5%)	36 (23.5%)	21 (13.7%)
Day time naps	23 (15.0%)	41 (26.8%)	24 (15.7%)	35 (22.9%)	30 (19.6%)

DST: Day time Sleepiness

**Table 3:** Correlation between sleep habits/problems, academic level, academic performance and sleep quality (PSQI score).

Variable	Sleep Quality	
	Correlation Coefficient	p-value
Academic level	-0.130	0.110
Usual time to wake up	-0.017	0.832
Bed time	0.136	0.093
Sleep latency	0.438	≤0.001*∗*
Drinking coffee	0.008	0.922
Snoring	-0.001	0.995
Nocturnal awakening	0.229	0.004*∗*
Nocturnal noise	0.277	0.001*∗*
Nightmares	0.132	0.103
Sleep-talking	0.117	0.151
Sleep-walking	0.244	0.002*∗*
Nocturnal awakening to use washroom	0.199	0.014*∗*
Morning tiredness	0.440	≤0.001*∗*
Daytime sleepiness during lectures	0.202	0.012*∗*
Daytime sleepiness during free time	0.149	0.066
Daytime naps	0.042	0.667
Academic performance	0.263	0.001*∗*
Living conditions	0.333	≤0.001*∗*
Leisure time	0.377	≤0.001*∗*

*∗*Correlation is significant at 0.05 level.

**Table 4:** Chi square test for association between PSQI assessed SQ and academic performance.

Self-reported Academic performance	Sleep quality (PSQI) n(%)		
	Good	Poor	Total
Excellent	19(63.3)	11(12.8)	30(19.6)
Good	34(45.3)	41(47.7)	75(49.0)
Satisfactory	14(31.1)	31(36.0)	45(29.4)
Poor	0(0)	3(3.5)	3(2)
Total	67(43.8)	86(56.2)	153(100)

Chi square =10.004, df =3, p=0.019*∗* [*∗*p-value <0.05] SQ: Sleep Quality

There was a significant positive association between self-reported academic performance and sleep quality as assessed by the PSQI (Table 4).

## Data Availability

All data used in this manuscript have been duly referenced.

## Appendix

### Glossary

  Excessive daytime sleepiness: a symptom of sleep disorders, characterised by difficulty in maintaining wakefulness during the day, and a tendency to fall asleep when seated or resting.
  Hypnagogic jerks: a sudden twitch often accompanied by falling sensation and a short awakening, commonly experienced just as a person begins to fall asleep.
  Sleep architecture: the structure and pattern of sleep, including overall sleep duration, sleep depth, sleep stages, and phases.
  Sleep cycle: the recurring progression of sleep stages repeated four or five times a night.
  Sleep deprivation: the condition of not having sufficient sleep.
  Sleep problems: any factor/medical condition which negatively affects healthy sleep patterns.
  Sleep gate: the point encountered during late evening when the circadian drive falls off and the homeostatic sleep drive becomes dominant allowing sleep to occur.
  Sleep hygiene: the practice of consciously adopting particular habits and routines in order to ensure a more sound sleep.
  Sleep inertia: the feeling of grogginess, impaired alertness, and decreased motor dexterity immediately following awakening.
  Sleep latency: the length of time taken for one to fall asleep.
  Sleep-wake cycle: the daily biological pattern of alternating sleep and wakefulness which is roughly 8 hours of night sleep and 16 hours of daytime wakefulness in humans.

## Abbreviations

DSM-IV: Diagnostic and Statistical Manual of Mental Disorders IV
IBM: International Business Machines
NREM: Nonrapid eye movement sleep
PSQI: Pittsburgh Sleep Quality Index
REM: Rapid eye movement sleep
SPSS: Statistical Package for Social Sciences
SSM: Sleep state misperception.
